# Supplementary material for: The effect of mobile text messages on knowledge and perception towards cancer and behavioral risks among college students, Northeast Ethiopia: A randomized controlled trial protocol
Source: PLoS One. 2021 Jul 9;16(7):e0253839. doi: 10.1371/journal.pone.0253839 (PMC8270214; doi:10.1371/journal.pone.0253839)
Supplement: S1 File — (DOCX) [file pone.0253839.s003.docx]

**The Effect of Mobile Text Message in Behavioral Risk Factor of Cancer among Governmental Collage Students, Northeast Ethiopia, A Randomized Control Trial**

**Principal investigator: Foziya Mohammed (PHO, MPH in Nutrition)**

**Co-investigators: Anissa Mohammed (MPH in Epidemiology and Biostatics)**

**Zinet Abegaz (BSC, MPH in RH)**

**A RESEARCH PROPOSAL TO BE SUBMITTED TO WOLLO UNIVERSITY RESEARCH COORDINATING OFFICE**

**September, 2019 Dessie, Ethiopia**

# Acknowledgment

It is grateful to thank the Department of Public Health, College of Medicine and Health Sciences, and Wollo University for allowing us to prepare this research proposal.

# Acronyms/ Abbreviations

| HBM | Health Belief Model |
| --- | --- |
| NCD | Non Communicable Disease |
| NHL | Non-Hodgkin lymphoma |
| ID | Identification |
| SPSS | Statistical Package for Social Science |
| SRS | Simple Random Sampling |
| VIF | Variance Inflation Factor |
| WU | Wollo University |

Table of Contents

[Acknowledgment II](#_Toc51228073)

[Acronyms/ Abbreviations III](#_Toc51228074)

[List of Tables VI](#_Toc51228075)

[List of figures VII](#_Toc51228076)

[Summary VIII](#_Toc51228077)

[**Introduction** 1](#_Toc51228078)

[Significance of the study 3](#_Toc51228079)

[Objective 5](#_Toc51228080)

[Methods 6](#_Toc51228081)

[Study area 6](#_Toc51228082)

[Study period 6](#_Toc51228083)

[Study design 6](#_Toc51228084)

[Source population 6](#_Toc51228085)

[Study population 6](#_Toc51228086)

[Sample Size Determinations 7](#_Toc51228087)

[Sampling procedure 7](#_Toc51228088)

[Inclusion and exclusion criteria 8](#_Toc51228089)

[Inclusion criteria 8](#_Toc51228090)

[Exclusion criteria 8](#_Toc51228091)

[Study Variables 8](#_Toc51228092)

[Dependent Variables 8](#_Toc51228093)

[Independent Variables 8](#_Toc51228094)

[Operational definitions 9](#_Toc51228095)

[Data collection procedure 9](#_Toc51228096)

[Outcome measures 10](#_Toc51228097)

[Primary outcomes 10](#_Toc51228098)

[Secondary outcomes 10](#_Toc51228099)

[Data quality control 11](#_Toc51228100)

[Data analysis procedures 11](#_Toc51228101)

[Dissemination of results 12](#_Toc51228102)

[Ethical consideration 12](#_Toc51228103)

[Work Plan 13](#_Toc51228104)

[Budget Plan 14](#_Toc51228105)

[References 15](#_Toc51228106)

#

# List of Tables

[Table 1: Work plan of the study 11](#_Toc20307064)

[Table 2: Budget break down 12](#_Toc20307065)

# List of figures

[Figure 1: Schematic presentation of sampling procedure 7](#_Toc20307551)

# Summary

**Background**: Cancer is an emerging public health problem in Ethiopia. A significant proportion of premature cancer deaths are preventable. The socio-economic impact of cancer can be considerably reduced provided that effective interventions are put in place to control risk factors. Text-messaging has been currently targeted as a simple and efficient tool for providing people with health information.

**Objective**: To evaluate the effectiveness of mobile text message in improving behavioral risks of cancer among college students

**Methods**: *Design*: a single-blind, 2-arm randomized controlled trial will be used. *Setting*: the study will be conducted among public colleges in Dessie town, Northeast Ethiopia. *Study population*: students who are studying in all public colleges. *Intervention*: a text message related with cancer risk factors once a day for two months. Control groups will receive general health related text message once a week for two months. *Data*: Socio-demographic, health belief variables and behavioral risk factor of cancer will be collected before and after intervention. Text message will be provided based on health belief model. *Primary outcomes*: cancer risk knowledge score and risk perception will be measured at baseline and 6 month post randomization. *Secondary outcomes*: a change in mean healthy diet score, physical activity level, alcohol intake and tobacco use will be measured at baseline-, 3, and 6 month post-randomization. ***Analysis***: We will compute descriptive statistics for each outcomes pre and post intervention. To test the change in score of HBM, paired t-test will be used. Analysis of Covariance will be used to test over group comparison.

**Work plan and Budget**: The study will be conducted from September 2019 to December 2020. The total required budget is estimated to be **95,243.4 ETB.**

# **Introduction**

Cancer is among the leading causes of premature death worldwide. In 2015, the incidence was estimated to be 17.5 million and was responsible for 8.7 million deaths globally [1]. Previously it was described as a disease of the high-income countries, but now it is also an important public health problem in low- and middle-income countries (LMICs). Lifestyle changes, rapid urbanization, cultural transition, and an increase in life expectancy could attribute to a rise in incidence. From 2005 to 2015, the highest (10–20%) increase in the incidence of cancer was observed in the World Health Organization (WHO) African region [2, 3]. Hence, it imposes an enormous burden on the already overwhelmed health care system of LMICs.

In Ethiopia, 5.8% of the total national mortality in 2015 was due to cancer [3, 4]. In 2015, the numbers of cancer cases were estimated to be 21,563 and 42,722 among males and females respectively [2]. Cancers of the breast, cervix, colorectal cancer, Non-Hodgkin lymphoma (NHL), leukemia, cancers of the prostate, thyroid, lung, stomach, and liver are the most commonly occurring cancers in the country [2-4]. Despite a rise in incidence, cancer still remains among the least public health priorities in Africa, mainly because of the immense burden of communicable diseases along with limited resources [5, 6].

The risk factors for cancer can be broadly categorized into four: behavioral, biological, environmental, and genetic. Behavioral risk factors include tobacco use, harmful use of alcohol, unhealthy diet and physical inactivity [4]. These lifestyle factors are potentially avoidable if appropriate preventive interventions are implemented [1, 7]. Human behavior is a key to the etiology of cancer and presents channels for targeted and sustained intervention [4, 8]. The effect of cancer can be significantly reduced through effective interventions to improve modifiable risk factors, early detection of cases, and appropriate management and care for those with the disease [3].

In Ethiopia cancer screening, diagnosis and management are sub-optimal and population-based data are limited to few cities [3]. Few studies in the country showed poor knowledge towards risk factors and screening were identified as important factors for poor utilization of cancer screening and other prevention services [6, 9, 10]. Thus, behavioral intervention might address a wide variety of key processes and outcomes across the cancer control continuum from prevention to care for survivors [8]. As part of strategy, text messages are currently considered to be the most feasible and widest-reaching mHealth intervention, as they do not require internet or any other advanced facilities [11]. Therefore, this study aimed to measure the effectiveness of text message to improve behavioral risks, which could be helpful to raise cancer awareness and promote cancer prevention and control in this country. We hypothesize that the mobile text messages will improve awareness and risk perception of cancer, ultimately improve the risk reduction behavior. Given the demonstrated effectiveness, delivering the intervention to a larger community might significantly reduce the public health burden of this disease

## Significance of the study

Developing countries including Ethiopia policies and programs are mainly focuses on communicable disease, but know a day’s developing countries are treated with cancer and other non- communicable disease more than communicable disease and face double burden of such problems due to urbanization and changing lifestyle of the community, are a severe threat to our economic development due to the long-term costs of treatment and the negative effects on productivity. Thus this study will give an input via measuring the effectiveness of cost effective mobile text message on reduction of preventable cause of cancer. Measuring the effectiveness of interventions is relevant for policy makers to choice appropriate and easily focus on interventions.

#

# Objective

Our study objective is to assess the effect of cancer specific daily mobile text messages, for early adults, on cancer risk knowledge and risk reduction behaviors such as healthy dietary habit, physical activity, reduction of alcohol consumption and quit smoking, compared to a general health message once a week, North east Ethiopia, 2019

`

#

# Methods

## Study area

The study will be conducted in all public colleges in Dessie town, the administrative town of South Wollo, Northeast Ethiopia. It is 401 kilometer far from Addis Ababa, the capital city of Ethiopia. There are three public colleges in the city, which provides several academic and training services. All public colleges in the city will be included, namely Woizero Siheen Polytechnic College, Dessie College of Teachers Education, and Dessie Health Science college. Woizero Siheen Polytechnic College was established in 1930 and currently provides technical and vocational education and training (TVET) in 5 different campuses: Main Campus (Siheen Campus), Merho Campus, Hotie Campus, Dawdo Campus and Menen (Vocational Campus). The College has more than 325 employees (including administrative and academic staff) and 5975 trainees in regular and night-shift programs. The college is also providing a short-term, non-formal and in-company training. Dessie College of teacher’s education was established in 1980 and has around 107 academic staffs and 1426 students. Dessie Health Science College is not included, as the students in this college expected to have knowledge on risks of cancer and preventive mechanisms.

## Study period

Intervention will be provided from April 01, 2020 to May 30, 2020 G.C.

## Study design

The study will use single blind randomized control trial study design that will assess the effect of mobile text message in behavioral risk factor of cancer among College students and utilize quantitative method of data collection.

## Source population

All Governmental Collage students will be our source population for the intervention and control group

## Study population

All Governmental Collage students will be also our study population for the intervention and control group

## Sample Size Determinations

The sample for this study will be determined using the assumption of superiority trial design to demonstrate the superiority of a new intervention or text message compared to the existing knowledge on cancer prevention. The following formula and assumption will be used to calculate the sample size [12].


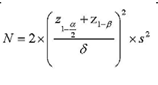


**Where:**

N=sample size per group;

**δ=**the mean difference of Perceived self-efficacy to adopting behavior between text message and exiting knowledge **δ= 2.9**

S^2^=SD= Pooled standard deviation of both comparison groups S= 6.22, S^2^= 38.74

z= 95% confidence level and 80% power
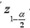
=1.96,
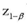
=0.845

The total sample size with 10% loss to follow up of study subjects is 160 (80 interventions and 80 controls) students will be selected randomly.

## Sampling procedure

Both Woizero Siheen Poly Technique Collage and Dessie Teachers Training collage will be included in the study. Sample size will be allocated based on proportional to population size. Students’ ID will be obtained from each and used as sampling frame. Then Simple random sampling method will be used to select eligible study subjects. After the baseline assessment, participants will be randomized to either usual care (control) or the text message intervention group in a uniform 1:1 (control: intervention) allocation ratio, using computer-based randomization service.

SRS

SRS

PPS

**160**

##

Figure 1: Schematic presentation of sampling procedure

## Inclusion and exclusion criteria

### Inclusion criteria

Having a personal (own) mobile phone and age >18years

### Exclusion criteria

Self-report of having any cancer, enrolled in another interventional study that use text message as a tool and Dessie Healthy Science Collage students will be excluded from study.

# Study Variables

## Dependent Variables

Behavioral risk factors of cancer

## Independent Variables

Socio-demographic variables: sex, age, marital status, religion

Health belief model (HBM) variables: Perceived susceptibility towards cancer, Perceived severity of cancer, Perceived benefits of adopting behaviors, Perceived barriers to adopting behaviors, Perceived self-efficacy for adopting dietary behaviors ,Internal cues for adopting dietary behaviors.

Others: Parental education, pocket money, collage, year of study, family history of cancer

## Operational definitions

**Intervention group:** a group who received daily cancer specific mobile text message.

**Controls**: comparative group participated in the study and received general healthy mobile text message once a week.

**Behavioral risk factors of cancer:** tobacco use, harmful use of alcohol, unhealthy diet and physical inactivity with a series of items will be used to measure the behavioral outcomes the items in this subscale will be measured on a Likert scale ranging from 1= “I never did” to 6 = “I did every day”.

## Data collection procedure

Self-administered questionnaire will be used to assess baseline and end line data of behavioral risk factors and other variables. To maintain blinding, randomization will occur after the assessment via computer, and then mobile text message will be delivered to participants. Participants in the control group will receive an initial text message welcoming them to the study, then received general healthy text message once a week. The intervention group will receive a text message support program, where they will receive seven messages per week at random times and days for two months. The text messages will be sent to participants in the morning. The principal investigator will be responsible to check the status of each sent item. If a message failed to be delivered, it will be sent again to the recipient. If delivery status keeps to be failed, phone call will be performed to discuss the issue and take measures accordingly. The messages will be prepared and delivered based on the health belief model (HBM). The focus of text messages will be on the prevention of behavioral risk factor of cancer such as harmful use of alcohol, unhealthy diet, tobacco smoking and physical inactivity.

### Outcome measures

All the primary and secondary outcomes will be measured at baseline and at 6 month post-randomization. Self-administered questionnaire will be used to collect the baseline and post-intervention socio-demographic characteristics, knowledge towards cancer, risk perception, and lifestyle behaviors.

### Primary outcomes

The primary outcomes for this trial are knowledge of behavioral risks of cancer and risk perception. To assess the risk perception of cancer, we will obtain data on the health belief model variables: Perceived susceptibility towards cancer ( e.g. You perceived that you will get risk of developing cancer at any time of your life), Perceived severity of cancer (e.g. “Cancer is fatal, and its complication are dangerous/severe in life”) Perceived benefits of adopting behaviors (e.g. “Having a healthy nutrition would decrease the probability of many type of cancers”) Perceived barriers to adopting behaviors (e.g. “My monthly income is insufficient to take the recommended diet for the prevention of cancer”), Perceived self-efficacy for adopting dietary behaviors (e.g. “I am confident that I can prevent cancer through healthy lifestyle”) Internal cues for adopting dietary behaviors (e.g. “when knowing the death of any by cancer, it flips me to perform the preventive behaviors”) the items of this subscale will be measured on a Likert scale ranging from 1= “Strongly disagree” to 5 = “Strongly agree”.

### Secondary outcomes

**Dietary habit**: will be measured using the food frequency questionnaire, the type and frequency of food items a participant consumed in the past 1 month.

**Physical activity level**: will be measured using International Physical Activity Questionnaires short form (IPAQ-S). The questions will ask the number of days and time participants spent doing vigorous physical activity, moderate physical activity, walking, and sitting in the last 7 days.

**Alcohol intake**: will be assessed using a tool adapted from the National Institute on Alcohol Abuse and Alcoholism (NIAAA), which we will use to assess participants consumption in the past 1 month.

**Tobacco use**: will be assessed using a series of questions adapted from the World Health Organization (WHO), which will assess the duration, quantity and frequency of smoking.

## Data quality control

Validity of the items will be measured by an expert panel of specialists in health education and nutrition. They will judge about the necessity and relevance of the scale items. To control the data quality all data collectors will be trained for two days. Supervisors will monitor data collection process and the principal investigator will visit frequently. The questionnaire will be first prepared in English language and translated to Amharic and again re-translated in to English by another person to check for consistency. 10% of the questionnaire will be pretested. Internal consistency will be checked using SPSS version 23 until the acceptable level reached. Completeness of the collected data will be checked during data collection by the supervisors and the principal investigators.

## Data analysis procedures

The data will be checked for completeness and consistency, then categorized, coded and entered using Epidata version 3.1 to minimize error. Then, the clean data will be exported to Statistical Package for Social Sciences (SPSS) version 23.0 for analysis; descriptive summary using frequencies, proportions, graphs and cross tabs will be used to present study results.

Before and after intervention liner regression will be used to assess the crude association between each variables and behavioral risk factors of cancer and to identify variables for consideration in multivariable models. Those variable <0.2 P-value will be entered to multi-variable Liner regression. Then multivariable analysis will be performed using multiple Liner regression to control the effect of confounding variables and to identify other predictors of behavioral risk factors of cancer. Multi-co linearity will be checked for variables which will selected for multivariable liner regression with the help of SPSS version 23.0 using Variance Inflation Factor (VIF). Finally, predictors with a P-value <0.05 will be considered statistically significant and B with 95% confidence interval will be used to describe association.

Normality of the data will be checked through Kolmogorov–Smirnov test. Student’s paired samples t-test will be used to test the within-group changes in terms of HBM variables and behavioural outcomes. The analysis of Covariance will used to make over-group comparisons. Data will be reported as mean ± SD. The significance level for all of the results will be presented at the P<0.05 level.

## Dissemination of results

Results will be disseminated to all wollo university community through presentation and hard copy. A copy of it will be disseminated to stakeholders such as Dessie collages and health offices. Possibly, it will be presented in the Ethiopian Public Health Association annual conference. An attempt will be made to publish the information in one of the reputable journal.

## Ethical consideration

Ethical approval for the research will be obtained from Wollo University, College of Medicine and Health Science Research Ethics Review Committee. Both collages will be informed about the study objectives through a letter that will be written from Wollo university research coordinating office.

Written consent will be obtained from each selected participant to confirm willingness. Honest explanation of the study purpose, description of the benefits and an offer to answer all inquiries will be made to the respondents. Affirmation will be given that they are free to withdraw or discontinue participation without any form of prejudice. Privacy and confidentiality of collected information will be ensured throughout the process; measures will be taken to ensure respect, dignity and freedom of each individual participating in the study. All information gained during the study will be kept strictly confidential.

# Work Plan

Table 1: Work plan of the study

| Activities | Time  2019 | | | | 2020 | | | | | | | | | | | |
| --- | --- | --- | --- | --- | --- | --- | --- | --- | --- | --- | --- | --- | --- | --- | --- | --- |
|  | September | October | November | December | January | February | March | April | May | June | July | August | September | October | November | December |
| Proposal writing |  |  |  |  |  |  |  |  |  |  |  |  |  |  |  |  |
| Proposal defense |  |  |  |  |  |  |  |  |  |  |  |  |  |  |  |  |
| Ethical clearance |  |  |  |  |  |  |  |  |  |  |  |  |  |  |  |  |
| Preparation of tools |  |  |  |  |  |  |  |  |  |  |  |  |  |  |  |  |
| training of data collectors and supervisors |  |  |  |  |  |  |  |  |  |  |  |  |  |  |  |  |
| Baseline Data collection |  |  |  |  |  |  |  |  |  |  |  |  |  |  |  |  |
| Intervention |  |  |  |  |  |  |  |  |  |  |  |  |  |  |  |  |
| Follow up for 4 month |  |  |  |  |  |  |  |  |  |  |  |  |  |  |  |  |
| End line data collection |  |  |  |  |  |  |  |  |  |  |  |  |  |  |  |  |
| Data entry and cleaning |  |  |  |  |  |  |  |  |  |  |  |  |  |  |  |  |
| Data analysis and result writing |  |  |  |  |  |  |  |  |  |  |  |  |  |  |  |  |
| Result submission and presentation |  |  |  |  |  |  |  |  |  |  |  |  |  |  |  |  |

# Budget Plan

Table 2: Budget break down

| **Baseline assessment** | | | **Working**  **Days** | | | | **Per diem** | | | | **No. of workers** | **Costs in ETB** | | | **Total** | | |  |
| --- | --- | --- | --- | --- | --- | --- | --- | --- | --- | --- | --- | --- | --- | --- | --- | --- | --- | --- |
| Perdiem for the researchers/PI | | | | 7 days | | | | | 192 | 3 | | | | 3x7x192 | | 4302 | | |
| Perdiem for supervisor | | | | 4 days | | | | | 192 | 2 | | | | 2x4x192 | | 1536 | | |
| Perdiem for trainer | | | | 7 days | | | | | 192 | 2 | | | | 2x7x192 | | 2688 | | |
| Perdiem for trainee | | | | 4 days | | | | | 192 | 6 | | | | 6x4x192 | | 4608 | | |
| Perdiem for data collectors | | | | 10 days | | | | | 192 | 4 | | | | 4x10x192 | | 7680 | | |
| Perdiem for data analysis and interpretation | | | | 10 days | | | | | 192 | 3 | | | | 4x10x192 | | 5760 | | |
|  | | | | | | | **Sub total** | | | | | 26,574 | | | | | |  |
| **End line assessment** | | | | | | | | | | | | | | | | | |  |
| Perdiem for the researchers/PI | | | | | 7 days | | | 192 | | 3 | | | 3x7x192 | | | | 4302 |  |
| Perdiem for supervisor | | | | | 4 days | | | 192 | | 2 | | | 2x4x192 | | | | 1536 |  |
| Perdiem for trainer | | | | | 7 days | | | 192 | | 2 | | | 2x7x192 | | | | 2688 |  |
| Perdiem for trainee | | | | | 4 days | | | 192 | | 6 | | | 6x4x192 | | | | 4608 |  |
| Perdiem for data collectors | | | | | 10 days | | | 192 | | 4 | | | 4x10x192 | | | | 7680 |  |
| Perdiem for data analysis and interpretation | | | | | 10 days | | | 192 | | 3 | | | 4x10x192 | | | | 5760 |  |
|  | | | | | | | | **Sub total** | | | | | 26,574 | | | | |  |
| **Follow up** | | | | | | | | | | | | | | | | | |  |
| Perdiem for the PI for follow up ,  and write up | | 60  Days | | | | | | 192 | | 3 | | | 3x60x192 | | 34,560 | | |  |
| Text message | | 60 Days | | | | | | 50 cent | | 100 | | | 100x60x50 | | 3000 | | |  |
|  | | | | | | **Sub total** | | | | | | | | 37,560 | | | | |
| 5% Contingency | **4535.4** | | | | | | | | | | | | | | | | | |
| Grand total | **95,243.4** | | | | | | | | | | | | | | | | | |

# References

1. Woldeamanuel, Y.W., B. Girma, and A.M. Teklu, *Cancer in Ethiopia.* The Lancet. Oncology, 2013. **14**(4): p. 289-290.

2. Memirie, S.T., et al., *Estimates of Cancer Incidence in Ethiopia in 2015 Using Population-Based Registry Data.* Journal of global oncology, 2018. **4**: p. 1-11.

3. Globocan, *Global cancer observatory.* International agency for research on cancer ,World Health Organization 2018.

4. ETHIOPIA, F.M.O.H., *NATIONAL CANCER CONTROL PLAN ,2016-2020.* FMOH, OCTOBER 2015.

5. Wondemagegnhu Tigeneh, A.M., Ayenalem Abreha and Mathwose Assefa, *Pattern of Cancer in Tikur Anbessa Specialized Hospital Oncology Center in Ethiopia from 1998 to 2010.* International Journal of Cancer Research and Molecular Mechanisms, 2015. **1.1**.

6. Abeje, S., A. Seme, and A. Tibelt, *Factors associated with breast cancer screening awareness and practices of women in Addis Ababa, Ethiopia.* BMC Women's Health, 2019. **19**(1): p. 4.

7. Haileselassie, W., et al., *The Situation of Cancer Treatment in Ethiopia: Challenges and Opportunities.* Journal of cancer prevention, 2019. **24**(1): p. 33-42.

8. Gotay, C.C., *Behavior and Cancer Prevention.* JOURNAL OF CLINICAL ONCOLOGY, 2005. **23**(2).

9. Getachew, S., et al., *Cervical cancer screening knowledge and barriers among women in Addis Ababa, Ethiopia.* PLOS ONE, 2019. **14**(5): p. e0216522.

10. Dibisa, T.M., et al., *Breast cancer screening practice and its associated factors among women in Kersa District, Eastern Ethiopia.* The Pan African medical journal, 2019. **33**: p. 144-144.

11. Cole-Lewis, H. and T. Kershaw, *Text messaging as a tool for behavior change in disease prevention and management.* Epidemiologic reviews, 2010. **32**(1): p. 56-69.

12. Dehdari, T., L. Dehdari, and S. Jazayeri, *Diet-Related Stomach Cancer Behavior Among Iranian College Students: A Text Messaging Intervention.* Asian Pacific journal of cancer prevention : APJCP, 2016. **17**(12): p. 5165-5172.
