# Supplementary material for: The effect of mobile text messages on knowledge and perception towards cancer and behavioral risks among college students, Northeast Ethiopia: A randomized controlled trial protocol
Source: PLoS One. 2021 Jul 9;16(7):e0253839. doi: 10.1371/journal.pone.0253839 (PMC8270214; doi:10.1371/journal.pone.0253839)
Supplement: Attachment — Submitted filename: Response to Reviewers.docx [file pone.0253839.s004.docx]

**Response to Reviewers**

PONE-D-20-05561
The effect of mobile text message on behavioral risk factors of cancer among college students, northeast Ethiopia: a randomized controlled trial protocol

On behalf of all authors, I would like to thank editors and reviewers for their valuable and constructive comments and revisions to improve the excellence of this paper. We revised the manuscript based on the comments. Here is point by point response for each comments and questions.

**Reviewer #1:**

This is a "Registered Report Protocol", where the investigative team plans to conduct a 2-arm randomized controlled trial (RCT) to test the effectiveness of mobile text messages in providing necessary health information for cancer prevention, compared to controls. While the study design is appropriate, I have some serious concerns on the actual science behind the study. In particular, I am not sure what an over-simplified RCT of this sort (spanning across only 6 months) will actually do, if any, to the cause. The intervention will be delivered, strictly, via text messages, to non-cancer (or, no prior diagnosis of cancer) subjects. There is no way to ascertain even whether the subjects are "actually reading/attending" the text messages, and responding to those (given that none of the subjects enrolled are actually cancer patients). Furthermore, following those for 6-months seemed to be a very short time to evaluate any effectiveness in that regard. In my opinion, all these will entail a "very pilot" study.

- **Response:** Thank you for your comment*.* The aim of the study is to test the effect of text message on changing knowledge, perception and behavior of unhealthy lifestyle which led to cancer. We agree that, as a digital public health intervention, it is not straightforward to ascertain whether participants read it or not. We can confirm whether the text is delivered, using the delivery report. We planned to assess the effectiveness under the assumption of the participants will read the text message, means, it is also part of the effectiveness. We also agree that 6 months may not be sufficient to evaluate effectiveness for a large scale intervention. However, this study will provide an indication whether the intervention has a potential for large scale intervention. Therefore, the study is a pilot effectiveness study. Based on the result we will scale-up to a large area with longer follow-up. Actually, 6 month could be sufficient to assess the change in knowledge and perception. However, the effectiveness on behavior change needs longer follow up period. If the perception is changed, we also expect a change in short term behavioral change. Hence, given the feasibility, the current protocol will pilot the effectiveness within 6 month.

Sample size/Power: The sample size/power statement should explicitly mention the statistical test used.

- **Response:** Thank you for the comment. The statistical test to be used is mentioned in the data analysis plan in line 215-230. The sample size with assumed power is described in line 131 of the revised version.

Randomization: From the randomization procedure described, it is not clear if this is a simple randomization, or a block. I would recommend a block strategy, which will ensure sample size are balanced across groups over time.

**Response**: Thank you for your suggestion. We will use a block randomization and we described it in line number 105.

Randomization: The authors need to mention how "Allocation Concealment", and "Blinding", considered two separate concepts, will be actually performed.

- **Response:** Blinding: All students will be told that, they will receive a text message from a specific mobile number and will not be told whether they are in the intervention or control group. Then a general health text message will be sent to the control group and the intervention text will be sent to the intervention group
- Allocation concealment: this will be done using SNOSE (sequentially numbered opaque, sealed envelope) technique when the students are enrolled in the study. The students’ ID will be put in envelop, then the investigators will randomize the intervention group accordingly.

Statistical Analysis plan: The design is longitudinal; so I was wondering why a small longitudinal analysis plan was not proposed. A longitudinal analysis (via mixed models, or generalized estimation equations) will allow one to have a better picture on how the trial actual progressed, and the subsequent estimates of the relevant parameters.

**Response:** Thanks for your comment. Given the randomization is appropriate, simple statistical tests could be sufficient. However, it is also possible to consider the dependency in measurements before and after the intervention. We accept your comments and we revised our analysis plan in line 215-230.

**Reviewer #2:**

• The bibliographic reporting system is incomplete. There is no reference within the text to the cited bibliography at the end.

**Response:** Thank you for your comment. We revised it accordingly

• In page 1: Department of epidemiology and biostatics … it’ ok… but it would be better to use the word biostatistics

**Response:** Thank you for your comment we have corrected it as suggested*.*

• In page 8: Epidata version 3.1: There is no relevant link. Moreover, it would be helpful to have a brief explanation, as a footnote, about using this software.

**Response:** We added the relevant link.

• in page 1: “Department of public health nutrition, ,…” with: “Department of public health nutrition, ….”

**Response:** We corrected it as suggested

• in page 4: “..which could be helpful to raise cancer awareness…” with: “..which could be helpful to raise awareness among citizens regarding cancer issues..”

**Response:** We modified it as suggested

**Reviewer #3:**

1. The authors plan to send a text message once a day for two months, which seems feasible. The daily messaging strategy should be effective given the relevant literature, but have the researchers done any kind of pilot testing to determine the appropriate amount of text messages for college students? They might become fatigued after receiving a message every day for two months and tune out the messages, especially since those in the treatment group are receiving messages about one topic (cancer risk).

**Response:** Thank you for your comment. It is true that the participants might be fatigued. To minimize it, we will frame the text messages to be brief, easy to understand and attractive.

2. Why are the participants in the control group only receiving one message per week and not one a day? Can the researchers also explain their rationale for measuring cancer risk knowledge and risk perception at baseline and six months post-randomization? Why not immediately after the two months?

**Response:** Thank you for your suggestion. The main reason to send once a week is feasibility reason. We will consider sending other healthy related messages for control groups daily for two month. It is possible to measure knowledge and perception at two month however, as the follow up duration is short, it will overestimate the effectiveness.

3. On page three, the authors state, “Behavioral risk factors include tobacco use, harmful use of alcohol, unhealthy diet and physical inactivity [4]. These lifestyle factors are potentially avoidable if appropriate preventive interventions are implemented.” I don’t disagree with this claim, but the authors should also consider the structural factors and a lack of access to resources in some cases that can prevent one from making lifestyle changes.

**Response:** Thank you for your suggestion. We agreed with your idea it is important to add such factors if feasible. However, in low-income countries, persons with full resource access could mostly practice unhealthy lifestyle because they can easily purchase tobacco, alcohol, frequently consume unhealthy diet (fried foods, meat, cream, butter, cake this are costly foods) and practice sedentary life style (no walk always use car for transportation). This all are considered as the manifestation of rich person. When we come up with our concern this factors are potentially avoidable if they know such factors lead to fatal outcome, even the poorer can also minimize these lifestyle factors.

4. The health belief model is mentioned at various points throughout the manuscript, but there is no background information presented in the literature review. There should also be a stronger rationale for why this framework was chosen for the current study.

**Response:** Thank you for your comment. We added a paragraph in the introduction section from line 77 to 82. The HBM is one of the most widely used theoretical frameworks for understanding health behavior. It is psychosocial model that is designed to help understand health behaviors which prevent disease, or detect disease when a patient has little, or no symptoms. Unlike other models used to describe and predict health behavior, such as theory of planned behavior, the HBM focuses on intra-personal factors, including risk related beliefs which influence individuals’ health related decision making. This is particularly important for this study as it aims to determine knowledge and risk perception of cancer.

5. There needs to be more information provided in the “recruitment and randomization” section. Based on the information here, it is difficult to determine if this study is feasible. Have the administrators from the universities already agreed to these protocols? Are there contracts in place with a service provider or third party who will be sending the messages? Who will write the messages? How will students opt in to the program? What is the procedure for opting out? How will students provide consent over text messaging?

**Response:** We added some sentences in “recruitment and randomization” section as suggested from line 144 to 154. We already agreed with each college officials. During baseline assessment we will receive each student’s phone number with ID and inform that they will receive text message in the coming two months. We will submit text messages to Wollo University research coordinating office who approved our protocol then they will send to students.

6. There also needs to be additional details provided about the messages. Are the researchers writing the messages? Are staff and faculty from the universities writing them? Will there be student/peer input so the language resonates with the college students? Will a messaging expert review these before they are sent out? It would also be helpful to see examples of the messages the researchers plan to use.

**Response:** Thank you for your suggestion. We revised it accordingly. The intervention message will be developed by a multidisciplinary team of oncologists, students from the college, nutritionist, and psychologists in iterative way. The message will be developed based on the American cancer society guideline for Diet and physical activity and SEOM clinical guidelines to primary prevention of cancer. Then, health education expert and nutrition specialist will revise messages, we mentioned it in “data quality control and management section” line 203 and 204. Finally, Wollo University research coordinating office will send messages with the supervision of investigators. When the program started, they will receive a phone call to inform that the message is delivering to them starting from now on wards. Daily, we will check delivery report and will assure that who will receive messages, if the participants not received messages they will receive phone call and resend it again. We add one example of text message in the “intervention section” line number 169 and 170.

7. “Primary outcomes” section, page seven- please cite the scales you plan to use to measure the health belief model variables.

**Response:** Thank you for your suggestion. We revised it as suggested.

8. Will the surveys be sent over the phones?

**Response:** Yes

The NIAAA measure for alcohol intake will be used to “assess participants’ consumption in the past 1 month.” However, this does not seem to align with the original timeline for collecting participant data.

**Response:** Thank you for your comment. One month is the time frame for the NIAAA, which is a standard tool. Both pre- and post- intervention measures will consider past one month. It is not align but we will measure its effect as one variable. Moreover, it is a standard tool which used to capture data on usual alcohol consumption unless we don’t know the participant take alcohol in the occasion.

9. On page eight, the authors make it sound as though the questionnaires will be administered in person. Is this the case? If so, how will responses be matched (baseline and post-test) and how will the researchers match responses with phone numbers/text message (control or treatment) group? There needs to be more details about how responses to the questionnaire items (pre and post) will be matched with participants in the text messaging groups. Also, can the authors explain why they are administering the questionnaires in person rather than through a survey link sent in a text message?

**Response:** We will measure it as a group as we will infer it to the population, and not concern the individual data which match at the baseline and the end. We will conduct paired t test to measure with in group variation and ANCOVA for over group variation.

The questionnaire include sensitive question in which the participant don’t disclose unless self-administered. Moreover, it is a part of ethics to maintain the participant’s privacy in providing unbiased data.

10. I would also like to see more information about the message content. The researchers mention on page nine that the messages will be “positively-framed” and “semi-personalized.” Can the researchers provide examples of these? Is there a communication specialist on this project who is writing and/or reviewing the messages in accordance with communication theory?

**Response:** health education expert and nutrition specialist will revise messages, we mentioned it in “data quality control and management section” line 203 and 204. We add one example of text message in the “intervention section”. (E.g. having unhealthy diet increases the risk of cancer in each person, so unhealthy food habits should be put aside) line number 169 and 170.
