# Supplementary material for: The effect of mobile text messages on knowledge and perception towards cancer and behavioral risks among college students, Northeast Ethiopia: A randomized controlled trial protocol
Source: PLoS One. 2021 Jul 9;16(7):e0253839. doi: 10.1371/journal.pone.0253839 (PMC8270214; doi:10.1371/journal.pone.0253839)
Supplement: Attachment — Submitted filename: Response to Reviewers.docx [file pone.0253839.s005.docx]

**Response to Reviewers**

PONE-D-20-05561
The effect of mobile text message on behavioral risk factors of cancer among college students, northeast Ethiopia: a randomized controlled trial protocol

PLOS ONE

On behalf of all authors, I would like to thank editors and reviewers for their continued constructive comments and suggestions to improve the excellence of this manuscript. We revised the manuscript based on the comments. We hope that we addressed all the comments and questions. Here is a point by point response for each comment and question.

**Editor:**

I would also suggest that you change 'text message' to 'text messages' in your title, as that is more grammatical.

**Response**: Thank you for your comment we modified it as suggested

**Reviewer #3:**

1. The authors did not fully respond to my first question. Did they complete a pilot study or collect some kind of preliminary data to determine the amount of messages to send? For this same point, can the authors explain what they mean by framing messages so they are "attractive"?

**Response:** Thank you for your comment. We agree that the volume and the content of text messages should be piloted. We developed some messages, but they need to be pre-tested before applying to the main trial. We will conduct a pilot test of messages in a similar group of population at Haiq Polytechnic College, a city 40 km far from Dessie. During the pilot test we will evaluate whether the messages are easily understandable, the amount and volume of the messages through discussion with the pilot participants and measures will be taken accordingly for the main trial. We said the messages ‘attractive’ means they should be good-looking and appealing. If text messages are framed in a wrong way, they will not deliver the correct content.

2. The authors did not do a sufficient job of elaborating on the HBM in the literature review. There is no explanation of the major constructs including barriers, benefits, efficacy, cues to action.

**Response:** Thank you for your suggestion. We added more descriptions on the introduction section from line 75 to 83 which explains components of HBM.

3. The inclusion criteria states that participants must be between 15-35. Can the authors provide a rationale for this large age gap? This is a wide range and the health behaviors of adults in their 20s/30s are likely to be different from individuals in their teens.

**Response:** As it is described in line 127-128 of the method section, the inclusion criteria is 18 to 35 not 15 to 35. We concur with your idea that there might still be variations. Nevertheless, in the Ethiopian educational system those who completed grade 10 are allowed to join technical and vocational colleges if they couldn’t achieve sufficient scores to join preparatory schools (grade 11 and 12). On the other hand adults also can join these technical and vocational colleges. Therefore, the age pattern in the colleges is mixed. Most probably, students in the same college, school or university behave in the same manner. However, as you said it well, we also expect variation in the health behavior. Thus, instead of excluding those aged 18 to 19 from the study, we preferred to include them and perform the subgroup analysis and compare the results. During the analysis, we will make this age group in one category.

4. The authors did include a sample message, but I would like to see more examples of the content that will be used in this study. Given that this is a messaging study, there needs to be more explanation of the actual messages and what they look like.

**Response:** Thank you again for your comment. We added more examples of text messages in the intervention section from line number 174 to 186 which include each behavioral risk factor of cancer and the components of HBM.
