# Supplementary material for: The effect of mobile text messages on knowledge and perception towards cancer and behavioral risks among college students, Northeast Ethiopia: A randomized controlled trial protocol
Source: PLoS One. 2021 Jul 9;16(7):e0253839. doi: 10.1371/journal.pone.0253839 (PMC8270214; doi:10.1371/journal.pone.0253839)
Supplement: Attachment — Submitted filename: Response to Reviewers.docx [file pone.0253839.s006.docx]

**Response to Reviewers**

PONE-D-20-05561R2

The effect of mobile text messages on behavioral risk factors of cancer among college students, northeast Ethiopia: a randomized controlled trial protocol

PLOS ONE

On behalf of all authors, I would like to thank editors and reviewers for their continued constructive comments and suggestions to improve the excellence of this manuscript. We revised the manuscript based on the comments. We hope that we addressed all the comments and questions. Here is a point by point response for each comment and question.

**Reviewer #3:**

1. The authors proofread the added content about the HBM within lines 75-83 since there are a few grammatical errors. For example, "believe" should be "belief."

**Response:** Thank you for your suggestion. We revised the suggested content and the whole manuscript for grammar and spelling.

2. The authors should also include at least one or two lines mentioning self-efficacy and cues to action given that these are variables within the HBM, but are not mentioned here.

**Response:** Thank you for your suggestion. In the introduction section lines 85 and 86 stated about self-efficacy. We also added more descriptions from lines 81 to 83 which explains self-efficacy and cues to action.

3. The authors should carefully proofread the messages that they plan to use, which are listed within lines 175-188. This is critical given that this is a messaging study. Additionally, although this is perhaps beyond the scope of this study, I recommend that the authors be careful about using messages like, "cancer is fatal had no certain cure" since this fear-based and might lead to the recipient tuning out the message completely. Also, the message, "although you are addicted to drink alcohol, you can decrease the amount and quit it through time” is not entirely accurate (and also needs to be proofread) since people who are addicted to alcohol cannot always decrease their consumption and/or quit over time. Finally, messages like, "avoid laziness!" might be seen as condescending and can produce a boomerang effect. Some of these messages also fail to consider structural/social determinants of health and put all of the emphasis on individual behavior change.

**Response:** Thank you for your suggestion. In fact, the messages will be translated and revised critically by experts before sending to the participants. We modified the sample messages as suggested.
