# Supplementary material for: The effect of mobile text messages on knowledge and perception towards cancer and behavioral risks among college students, Northeast Ethiopia: A randomized controlled trial protocol
Source: PLoS One. 2021 Jul 9;16(7):e0253839. doi: 10.1371/journal.pone.0253839 (PMC8270214; doi:10.1371/journal.pone.0253839)
Supplement: Attachment — Submitted filename: Response to Reviewers.docx [file pone.0253839.s007.docx]

Response to Reviewers

PONE-D-20-05561R3

The effect of mobile text messages on behavioral risk factors of cancer among college students, Northeast Ethiopia: a randomized controlled trial protocol

PLOS ONE

First of all, on behalf of all authors, I wish to thank the editor and the reviewers for their valuable and constructive comments to improve the excellence of the manuscript. The manuscript went through a long review process since February 2020 and was reviewed by three reviewers. We revised it thoroughly and addressed all issues raised by reviewers. You can refer all the versions of the manuscript and response to reviewers. Due to the delay from the review process, we couldn’t start the intervention yet. Now, we are under pressure from Wollo University, the institution which partially funded the project, to start implementation and finalize the output. It’s taking unacceptably long time for just the protocol. We also consider the issues raised in the current review very important and we revised the manuscript based on the comments and issues raised by the editor and the reviewers for one last time. We are confident that we have addressed all these comments adequately and we therefore hope that the manuscript is accepted for publication. We kindly request the final decision for the manuscript. Find hereunder a point by point reply to the comments and questions raised by the editor and all reviewers.

**Editor**

1. Please review your reference list to ensure that it is complete and correct. If you have cited papers that have been retracted, please include the rationale for doing so in the manuscript text, or remove these references and replace them with relevant current references. Any changes to the reference list should be mentioned in the rebuttal letter that accompanies your revised manuscript. If you need to cite a retracted article, indicate the article’s retracted status in the References list and also include a citation and full reference for the retraction notice.

**Response:** Thank you for your suggestions. We have reviewed the manuscript particularly the reference list to meet the journal requirements and we hope that the manuscript in its current form does so. We did not use any papers that are retracted.

**Reviewer # 3**

1. I appreciate the authors' efforts to revise the manuscript and for their attention to my comments. I "accept" the manuscript in its current form and the authors have addressed my previous concerns. However, I want to reiterate the importance of having the messages reviewed by communication/messaging experts who have experience with text-messaging studies aimed at health behavior change, so that they can offer suggestions for framing, wording, etc. This study has the potential to make a unique and important contribution to the text-messaging/mobile health literature. I look forward to reading the findings once this is implemented and the data is analyzed.

**Response**:

Thank you for your constructive comments throughout the review process. The manuscript improved a lot from your feedback. As you suggested, once the protocol is accepted, we will start developing intervention tools (messages) in consultation with experts in communication and health education.

**Reviewer # 4**

1. As students in the same college are individually randomized to the intervention and control arms, there’s a large risk of contamination (e.g. students may simply compare the text messages received). Due to the target population and setting of interest, this study would have benefitted from the use of a cluster RCT design. However, as recruitment has already been completed, this should at the very least be discussed as an important limitation of the study design. Linked to this, as recruitment is already completed, I don’t see how this can usefully be published as a Registered Report Protocol, as there’s insufficient scope to improve on the study design.

**Response**:

Thank you for your comment. We agree that there could be intervention contamination in the trial. We have been discussing this issue within the team. However, due to the huge variation in the nature of colleges (technical vs teachers’ education college), cluster randomization will lead to selection bias. As a result, we decided to use individual randomization and instruct individual participants on the trial process thoroughly. We will discuss further the limitations of the study during the result report. We conducted a preliminary assessment and are now waiting for the protocol to be accepted before starting implementation of the intervention.

2. When setting up the rationale for the study in the Introduction, a better lay of the land is needed. It would be useful to know the proportion of adults and/or students in Ethiopia with access to mobile phones. There’s a vast literature on the effectiveness of text messages for smoking cessation, physical activity, etc. (please see a suggested reference to a review of systematic reviews below). It would be important to refer to this wide literature in the Introduction, that positive effects on behaviour have been observed, and then state that few existing interventions have been evaluated in LMICs, including Ethiopia (off the top of my head, I’m only aware of a couple for smoking cessation, with one in China and one in Vietnam):

<https://www.ncbi.nlm.nih.gov/pmc/articles/PMC4406229/>
<https://pubmed.ncbi.nlm.nih.gov/26730560/>
<https://www.ncbi.nlm.nih.gov/pmc/articles/PMC7381024/>

**Response**:

We do not have data on the coverage of mobile phones for the general population in Ethiopia. Nevertheless, our preliminary assessment indicated that almost all college students have access for it. Thank you for your suggestion of articles on the effectiveness of mobile text message on smoking cessation and health. We incorporated some of the articles in lines 97 to 99 of the introduction section. However, as far as we are aware, there is no study that evaluated the effectiveness in Ethiopia. We are very much grateful if you provide us with the link.

3. Although it’s a compelling aspect of the study that the intervention has been developed on the basis of the Health Belief Model, it’s unclear how the intervention messages map onto the different components of the HBM. It would be useful to make this more explicit, for example by including a table with example messages and indications in a second column as to how each message maps onto the different components of the model. At present, it’s unclear whether all components were covered by the messages. This is important to understand, as we wouldn’t expect the intervention to have an effect on, for example, self-efficacy, if the intervention messages themselves don’t directly attempt to boost self-efficacy.

**Response**:

Thank you for your suggestion. We didn’t start the intervention yet. Once the protocol is accepted, we will proceed developing messages in collaboration with experts in health education. As this is a protocol, we provided sufficient sample messages for each component of HBM in table one of the manuscript.

4. It’s unclear why participants aren’t recruited on the basis of engaging in one or multiple unhealthy behaviors? There’s arguably no need to raise awareness that excessive alcohol consumption is a risk factor for cancer in those who don’t engage in heavy drinking. It’s also unclear why the primary outcome isn’t behavior change – this would be of greater interest to the public health community and a more typical outcome for an RCT.

**Response**:

People might not be involved in heavy alcohol drinking or smoking at the moment. However, it does not necessarily mean that they have good knowledge on the risk of alcohol consumption and smoking on cancer. If people are not involved in unhealthy lifestyles, having good knowledge would help them to continue with such a healthy lifestyle. Those who don’t have good knowledge on the risk of physical inactivity, unhealthy dietary habits, smoking, and excessive alcohol consumption might be involved in such lifestyle sometime later. Therefore, our target group is not necessarily those who are practicing an unhealthy lifestyle. Nevertheless, we have planned to recruit more samples and take those with knowledge scores below two-third will be randomized. Our primary outcome is knowledge on cancer and lifestyle risk factors. We think that the reviewer did not realize the context of Ethiopia that people are not aware of behavioral risks of cancer. A significant proportion of the population still consider cancer as a disease related with bad spirits. Hence, it’s crucial to improve the knowledge before the actual behavior. Moreover, as our project is for 6 months duration, the effect on the change in behavior might not be large. In general, we will see the effect of our intervention on knowledge as primary outcome and assess behavior on four major risks as secondary outcome. Based on our result, we will consider the change in behavior in the scale up project for a longer duration.

Specific comments

Introduction

5. The second sentence about cancer incidence and mortality refers to global cancer statistics but the citation refers to cancer rates in Ethiopia. Please change to a more suitable reference, e.g.

<https://acsjournals.onlinelibrary.wiley.com/doi/full/10.3322/caac.21660>

**Response**:

Thank you for your suggestion. We revised accordingly.

6. It would be useful to more explicitly state that although there are many different risk factors for cancer (e.g. behavioral, biological, environmental), not all of these are modifiable, and that this study focuses specifically on modifiable health behaviours. Environmental exposure to, for example, air pollutants, and soil and drinking water contaminants, could theoretically also be addressed via behaviour change (although may require wider policy changes to impact behaviour).

**Response**:

We agree that environmental factors are also among the potentially modifiable risk factors. We revised it on page 68 to 69 of the revised manuscript. However, our main interest in this intervention is to improve knowledge, perception and behavior (to some extent) towards lifestyle risks of cancer. It is not possible to address every aspect of cancer risk factors with this short period and limited funding. Probably, we will consider to look at these issues in the follow up project.

7. Line 74 – there are some typos here, e.g. “The” is missing at the beginning of the sentence; “health related” rather than “healthy related”.

**Response**:

Thank you for your suggestion. We revised accordingly.

8. Line 85 – “and by their confidence that the recommended action” – this is repeating information on line 79; please remove.

**Response**:

Thank you for your suggestion. We revised it accordingly.

9. Line 86 – “Thus, we will use the HBM to measure the perception…” – this doesn’t fully elaborate on the study aims, which also includes the examination of behaviour change. Given this is described in the next paragraph, I’d suggest changing this to a broad statement that the HBM will therefore be used to develop an intervention to increase awareness of and perceived susceptibility to cancer, and affect change to four key health behaviors.

**Response**:

Thank you for your suggestion and we revised it accordingly.

10. Rather than stating both hypotheses and objectives for the study, I’d suggest including a list of the key research questions and how they relate to the different follow-up assessments and outcome variables of interest. For example, the first research question may be whether participants allocated to the intervention arm, compared with those allocated to the control arm, have increased knowledge of the cancer risks of smoking/alcohol/etc at a 6-month follow-up. Please list all of the main research questions and end points.

**Response**:

As suggested, we added the main research questions. However, the primary and secondary end points are sufficiently described in detail in lines 199 to 227 of the method section. We think that putting the end points again is no more than a repetition of primary and secondary outcomes.

Methods

11. Although a CONSORT flow chart is included, it would be useful to reference the CONSORT checklist in the ‘Study design’ section and mention that this was used in the design and reporting of the study: <http://www.consort-statement.org/>

**Response**:

We cited the reference and thanks for the suggestion.

12. The study setting section describes the number of staff members but the eligibility criteria state that only students are eligible to take part – it’s unclear why staff members aren’t also eligible? Also, it’s unclear why only those aged 18-35 years are eligible to take part? Please provide a rationale and remove any superfluous information about the employees in the ‘Study setting’ section (as they aren’t part of the target population).

**Response**:

Our aim is to see the effectiveness on students, which are more or less homogeneous groups. The staff and students are somehow different in terms of lifestyle and socioeconomic characteristics, which is not our interest to observe in this study. Hence, we removed the information about the number of staff as suggested.

13. The authors state that the study is powered to detect a difference in “knowledge and perception of cancer risk” but then go on to state that the power calculation is based on perceived self-efficacy – these are not identical psychological constructs, so it would be important to clarify which of these is the primary outcome for the study. It would also be useful to contextualise the 2.9-point expected difference in knowledge or self-efficacy – is this on a 5-point or 7-point Likert scale?

**Response**:

We didn’t calculate power, we just determined (assumed) 80% power to detect a 2.9 mean difference. Nevertheless, we corrected the editing problem that we calculated the sample size considering the self-efficacy. We will use 5-point Likert scale.

14. It’s unclear what this means “Sample size will be allocated to…” – does this mean only two colleges will be used for recruitment? If so, this needs to match the above ‘Study setting’ section.

**Response**:

There are three colleges in the town. One of them is Health Science College, which will be excluded as students from Health Science College are expected to have a different (higher) knowledge related to cancer and risks. Thus, the sample size will be allocated to the two remaining colleges (Woizero Siheen Polytechnic College and Dessie College of teacher’s education).

15. “Students’ IDs will be obtained…” – do you mean that all students with an ID will be contacted and asked if they are willing to join the trial?

**Response**:

As we clearly described, the students’ ID number will be used for random sampling (line 152 to 154). The confusion may be using the term ‘ID’ instead of ‘ID number’ and we modified it.

“*Students’ ID number will be obtained from the registrar's office of each college and used as a sampling frame. Then; a simple random sampling method will be used to select study subjects*.”

16. “Then; simple random sampling method will be used to select eligible study subjects” – this sounds incorrect. Surely interested students will be assessed for eligibility via a screening survey, with eligible participants randomized with simple randomisation to the intervention or control arm?

**Response**:

The simple random sampling here is not ‘the randomization to intervention and control arm’. Rather we are referring to the random sampling to be included in the study since we won’t enroll all students. From all eligible participants, we will select random samples for the study. Then, we will randomize either to the intervention or control arm using simple randomization (see lines 160 to 163 of the recruitment and randomization section). We removed the word ‘eligible’ to avoid confusion.

17. Blinding – it should also be stated that the researchers will be blinded to the allocation sequence (i.e. single blind).

**Response**:

We will use single blinding, in which only the study participants will be blinded. In such kind of interventions, it’s not feasible for investigators to be blinded. If investigators are also blinding, it is double blinding. Furthermore, the most important thing is not using the term ‘single’ or ‘double’ rather the description of who will be blinded (<https://www.bmj.com/rapid-response/2011/10/31/what-single-blind-trial>). We clearly mentioned in line 176 to 178 that study participants will be blinded to the intervention arm.

18. Control – please provide a bit more information on what is considered a ‘general health message’ in this context. I assume these won’t mention the four health behaviours of interest?

**Response**:

General health messages are contents other than the four mentioned behaviors and not related to cancer and the four lifestyle risks. The focus for the general health messages will be mainly related to infectious diseases such as sanitation, how to prevent HIV and TB, etc.

19. Primary outcomes – please comment on whether the 5-point Likert scale has been validated.

**Response**: it is validated in the article https://www.ncbi.nlm.nih.gov/pmc/articles/PMC5454653/

20. Secondary outcomes – please provide references to the specific food frequency questionnaire that will be used and whether it has been validated, the IPAQ-S and whether it has been validated, the NIAAA tool to assess alcohol consumption and the WHO tool to assess smoking. I’d recommend using the AUDIT or AUDIT-C for alcohol: https://apps.who.int/iris/handle/10665/67205 It’s unclear whether the smoking assessment will examine cigarette smoking only or any kind of tobacco?

**Response**:

The IPAQ short is internationally validated in different context including low income countries.

For tobacco use, we will consider any kind of tobacco.

Thank you for your suggestion. We will use AUDIT to assess alcohol consumption.

We revised and cited each tool accordingly.

21. The proposed statistical analysis plan looks good.

**Response**:

Thank you.

**Reviewer #5**

Dear PLOS ONE Editors,

Thank you for the opportunity to review an article entitled “The effect of mobile text messages on behavioral risk factors of cancer among college students, Northeast Ethiopia: a randomized controlled trial protocol” prepared by Hussien and colleagues.

This manuscript has already undergone peer review. It is well written and provides the relevant information for a trial protocol. However, I have a number of suggestions for the authors to consider for how to improve the proposed intervention and the trial, if still possible.

1. The authors selected Health Belief Model (HBM) as a theoretical basis for the intervention. The constructs specified by HBM can contribute to risky behaviours and behaviour change (e.g. beliefs about what are risk factors), but they primarily focus motivational factors. However, there are other important determinant of behaviour change – as mentioned by other reviewers earlier (e.g. social determinants). For example, another model of behaviour – Capability, Opportunity, and Motivation (COM-B) shows that for any behaviour change to take place one not only needs motivation, but also capability (knowledge, skills) as well as opportunity (social and physical). Therefore, an intervention that only target some of the COM-B elements without addressing other known barriers is likely not to be sufficiently effective. The evaluation survey is accounting for it (e.g. by asking about income sufficient to change behaviour), but it does not seem that the intervention suggest ways of changing behaviour when one has insufficient income. Can the authors still consider and address the limitations of HBM and if the intervention content could still be improved?

**Response**:

Thank you for the suggestion. As we described in lines 201 to 203, socioeconomic characteristics will be assessed and be considered to adjust using GEE in the analysis section. Regarding (COM-B), it is not our aim in this study to look at it. The COM-B model of behavior is to identify what needs to change in order for a behavior change intervention to be effective. Our aim is not on the process, rather on the outcome. The main reason to conduct a randomized study is to control for some of the important confounding variables. Once the most important variables are being adjusted, the rest residual confounders will be expected to be controlled in the randomization. Furthermore, the change in behavior is the secondary outcome, the primary end point of this study is change in cancer related knowledge.

2. I see some further potentially missed opportunities for increasing the effectiveness of the intervention. For example, important and effective components (or behaviour change techniques) of behaviour change interventions are setting goals, monitoring and feedback. Without specifying goals for behaviour change early on for the participants it will be quite improbable that they will engage with the intervention and attempt behaviour change (the attempts being a first step to change the behaviour).

**Response**:

We agree that components including setting goals, monitoring and feedback are crucial in behavioral change interventions. However, incorporating such components is not feasible in this short term study using mobile text messages. We cannot address everything in a single short term study. Our main aim is whether sending cancer specific text messages improve the knowledge (primary), perception and practice (secondary ) towards behavioral risks of cancer. Once again, our primary outcome is not a change in behavior. We will observe the change in behavior as a secondary outcome.

3. The intervention targets are very broad (and address a number of health behaviours) – ideally the content of the interventions would be tailored to the risk behaviours of that individuals to increase the relevance of the intervention. Previous SMS texting interventions that target a single behaviour (e.g. smoking cessation) were very complex and detailed, and it is unlikely that only a few sms text about one behaviour will be sufficient to change that behaviour.

**Response**:

We agree that a change in behavior needs a more comprehensive and multi-component intervention considering various aspects of complex human behavior. However, our main outcome is change in knowledge and perception. We will evaluate the change in behavior as a secondary outcome. We clearly elaborated the primary and secondary endpoints in page 8 and 9 of the method section.

4. Students in public colleges are more educated and technology-savvy group in the population. Additionally, they may also be at lower risk of engaging in some of the risky behaviours, and given their young age their cancer risk is also much lower. It would be important for the authors to clarify the benefit of testing this intervention among this population, rather than among adults in the general population for whom it may be more relevant and who may benefit more from it.

**Response**:

The main reason is, for a text message intervention participants should be able to read and write and have basics of mobile use. Hence, we will assess the feasibility and practicality to scale up this intervention to the general population in the future if it is effective among college students.

5. Additionally, the eligibility criteria do not specify that the participants should engage in any unhealthy behaviour. It is therefore possible that all of the participants will lead a healthy lifestyle and have no unhealthy behaviours. This is another important limitation of the study that will make it more difficult to show any effect of the proposed intervention. Can the authors consider to first screen participants for unhealthy behaviours and recruit only those who exhibit behaviours and the levels of these behaviours (e.g. smoking, high alcohol consumption, etc) that can realistically show any reduction or improvement during the trial?

**Response**:

We think that there is mis-understanding of our primary aim. Our primary outcome is to see the change in cancer related knowledge and perception. We will observe the change in behavior as a secondary outcome. From our previous experience, it is unlikely that all of the participants have no unhealthy behaviors. Furthermore, not being involved in unhealthy behavior does not necessarily mean that they have good knowledge on cancer. Having good knowledge would help those with unhealthy lifestyles to change and those with a healthy lifestyle to persist in that behavior. Hence, our target groups are all college students, not specifically those involved in unhealthy lifestyles.

6. Texting has been shown to be a cost-effective medium to deliver education or more complex behaviour change interventions for a number of health risk behaviours. Texting as a technology can have more reach than newer technologies (e.g. smartphones app) as they can be delivered on any mobile device. However, sms texts can be ignored, the options for content are very limited, and there is little or no possibility to assess engagement with the texts objectively. I encourage the authors to include more discussion around the pros and cons of using texting, and how some of the limitations for the interventions and evaluation will be addressed.

If the authors are no longer able to change the intervention or the study design, then it would be very helpful for the published trial protocol to include a discussion of these important limitations and highlight the contribution that this study still brings despite the known limitations.

**Response**:

Thank you for your suggestion. We added as suggested from line 307 to 310. Moreover, whether the participants looked at the texts and how frequent they checked for their texts will be assessed post-intervention. Thus participants’ engagement will be assessed later and be discussed in the limitation of the study results.
