# Supplementary material for: The effect of mobile text messages on knowledge and perception towards cancer and behavioral risks among college students, Northeast Ethiopia: A randomized controlled trial protocol
Source: PLoS One. 2021 Jul 9;16(7):e0253839. doi: 10.1371/journal.pone.0253839 (PMC8270214; doi:10.1371/journal.pone.0253839)
Supplement: Attachment — Submitted filename: Response to Reviewers.docx [file pone.0253839.s008.docx]

Response to Reviewers

PONE-D-20-05561R3

**The effect of mobile text messages on knowledge and perception towards cancer and behavioral risks among college students, Northeast Ethiopia: a randomized controlled trial protocol**

PLOS ONE

On behalf of all authors, I would like to thank the editor and reviewer for their valuable and constructive comments to improve the excellence of this paper. We revised the manuscript based on the comments. Here is a point by point response for each comment and questions.

**Editor**

As you will see from the reviewer comments below, only very minor further revisions are requested, namely 1) to change minor inaccuracies in the manuscript and 2) to add some of the justifications provided in the responses to reviewers regarding decisions on study design in the actual manuscript.

**Response:** Thank you for your suggestions. We addressed all comments and looking forward your prompt response.

**Reviewer # 4**

The authors have done a fine job addressing the reviewer comments. I only have two remaining points.

**Response**: Thank you

In the abstract, "To test the change in score of HBM" is unclear and should be changed to "To test the change in knowledge and perception" or similar.

**Response**: Thank you for your suggestions. We modified it in line 44 of the revised manuscript.

The 'Trial status' section still says that "We are currently recruiting and enrolling students in the study. As of February 2020, we have enrolled 160 participants." Given the authors' response that the intervention is yet to be developed and delivered, please amend this accordingly.

**Response**:

Thank you for your suggestion. We amended it in line 308.

**Reviewer #5**

I would like to thank the Authors for responding in detail to my suggestions and questions. It is very helpful to see it clarified that the primary outcome are changes in knowledge/perceptions, rather than behaviours. I accept most of the arguments put forward by the Authors and understand the urgency to conduct this research as per the original protocol and within the limits posed by the resources available.

**Response**: Thank you

I believe that the manuscript would be strengthened if the Authors incorporated some of the arguments from their latest response into the methods and the discussion section of the manuscript, i.e. to offer an additional justification (and a discussion of the limitations) for the different decisions made regarding study design.

**Response**:

Thank you for your suggestion. We incorporated major arguments which could improved the excellence of the paper in methods section from lines 121 to 122, and the discussion section in lines 284, 285, and 303 to 306.
